# Polymerization of aniline hydrochloride in reverse of microemulsion by batch and semicontinuous process using ionic and nonionic surfactants

**DOI:** 10.1080/15685551.2022.2063011

**Published:** 2022-04-12

**Authors:** G. Pier Villegas, A.G. Alvarado Mendoza, L.G. Guerrero Ram\u00EDrez, L.C. Rosales-Rivera, J. Aguilar, F.J. Moscoso S\u00E1nchez

**Affiliations:** a Departamento de Química, Centro Universitario de Ciencias Exactas e Ingenierías, Universidad de Guadalajara, Guadalajara, Jalisco, México; b Departamento de Ingeniería Química, Centro Universitario de Ciencias Exactas e Ingenierías, Universidad de Guadalajara, Guadalajara, Jalisco, México; c Departamento de Ciencias Tecnológicas, Centro Universitario de la Ciénega, Ocotlán, Jalisco, México

**Keywords:** Polyaniline, inverse microemulsion, nanoparticle, ionic surfactant, nonionic surfactant

## Abstract

The polymerization of aniline hydrochloride by inverse microemulsion in a batch process and the semicontinuous process was studied as a function of the surfactant ionic and nonionic. Polymerizations were carried out at 60°C for 4 h with a yield polymer of circa 67 and 27% wt. for ionic and nonionic surfactants. The conductivity of synthesized polyaniline by the semicontinuous process is higher up to three orders of magnitude than that of the batch process for both surfactants. The calculating degree of oxidation by UV-Vis showed the relative intensities of the quinoid to benzenoid unit around one. The morphology was determined by Scanning Electron Microscopy (SEM) and observed that the formation of the different morphologies is due to the self-assembly behavior of surfactant. The diameter z-average particle size (Dz) was studied by Transmission Electron Microscopy (TEM), which determined that the diameter particle in a semicontinuous state is larger than the one produced in a batch; this is due to the control of monomer addition in the system. These findings suggest that the polymerization process and the type of surfactant influence the properties of polyaniline.

## 1. Introduction

Polyaniline (PANI) is a material of great interest in the application of different technology areas, for instance: electronic devices, light-emitting diode (LED), rechargeable batteries, supercapacitors, and solar cells. This is due to its electrical conductivity and environmental stability [1,2]. The polymerization of PANI depends on the various factors that affect the viscosity properties, conductometry, and yield. Some of these factors are aniline/oxidant agent mole ratio, concentration, the nature of protonic acid, time of reaction, reaction temperature, and polymerization technique [3,4]. There is much research in the literature, but the oxidation of aniline in acidic aqueous media using ammonium peroxydisulfate as an oxidant has been mostly used [5–7]. For another part, PANI has been synthesized by heterogeneous polymerization emulsion, nanoemulsion, and microemulsion [8,9]. One of the few synthesis methods used is microemulsion polymerization. Microemulsions are homogeneous pseudoternary dispersed systems, they are constituted by two immiscible liquids (oil-to-water), appropriate amounts of surfactant, and/or co-surfactant. They are within thermodynamic equilibrium and can take on several microstructures such as oil in water (O/W) or water in oil (W/O) depending on temperature, composition, surfactant type, etc. [10,11]. A variant of the batch microemulsion synthesis method is semicontinuous polymerization, which can be used to obtain polymeric nanoparticles with defined morphology. The synthesis begins from a surfactant solution without the presence of a monomer. There are only empty micelles present at the beginning of the reaction and then the monomer is added continuously at a constant rate (Ra) [12]. On the other hand, numerous reports are available depicting the behavior and the physicochemical properties of microemulsions using surfactant ionic and nonionic [4,13,14]. However, there is little bibliography that describes using of microemulsions inverse [15], with surfactant ionic [16] and nonionic in polymerization semicontinuous for obtaining the conductive polymers [17]. The present contribution deals with the study of the polymerization of aniline hydrochloride through inverse microemulsion by the batch process and semicontinuous process as a function of the surfactant ionic and nonionic. The effect on the yield, particle size, structure, and conductivity of polyaniline was analyzed.

## 2. Experimental section

### 2.1. Reagents

Aniline hydrochloride monomer was synthesized by using 99% pure Aldrich brand reagent grade aniline (St. Louis, MO, USA), previously distilled at 185°C. The aniline hydrochloride salt was washed with 99% pure Wohler brand reagent grade ethyl ether (Iztapalapa, Mexico). Brand Aldrich ACS grade (St. Louis, MO, USA) ammonium persulfate (APS) was used as the oxidizing agent. Sodium bis(2-Ethylhexyl) sulfosuccinate (AOT) and polyethylene glycol sorbitol hexaoleate (PEG-SOR HEX) were used as anionic surfactant and nonionic both with HLB of 10 from Aldrich (St. Louis, MO, USA). Nitrogen gas with 98% pure from Infra de Occident (Guadalajara, Mexico) was used to purge and evacuate the oxygen content of the solution, and hydrochloric acid from Aldrich (St. Louis, MO, USA) was also used without purification. Selectro Pura brand deionized water (Guadalajara, Mexico). Dimethyl sulfoxide brand Aldrich grade ACS (St. Louis, MO, USA) was used as the solvent

### 2.2. Methodology

The synthesis of aniline hydrochloride (AHCl) was carried out by mixing equimolar amounts of previously distilled aniline and hydrochloric acid under stirring within a 100 mL round flask cooled with ice and water. As the solution cooled down, the formation of aniline hydrochloride salt crystals began. Once this process was finished, the salt was deposited in a separation funnel and washed with anhydrous ethyl ether. The salt was collected and dried in an oven at 50°C and stored for the next process. To make the phase diagram, compounds were weighed and introduced into glass ampoules, which were sealed and immersed in a temperature-controlled bath at 60°C until the system achieved the equilibrium of thermic stabilization. The samples were visually examined using a laser beam of light scattered as it passes through glass ampoules containing diluted microemulsion. If the effect of scattered light occurred, it was considered a microemulsion. Different recipes were tried for the synthesis and were chosen the compositions were more stable for realization. The first compositions have been 83.4%wt cyclohexane, 13% wt. AOT ionic surfactant, and 3.537% wt. AHCl into an aqueous phase. In the aqueous phase, the proportions were 3.074% wt. water, 0.342% wt. of AHCl. and 0.12% wt of APS. The composition second was 83.32% wt. cyclohexane, 10.78% wt PEG-SORHEX nonionic surfactant, and 5.90% wt. AHCl into an aqueous phase. In the aqueous phase, the proportions were 4.293% wt. water, 1.49% wt. of AHCl, and 0.117% wt. of APS. Both syntheses were made in a batch process and semicontinuous process. First, the surfactant AOT or PEG-SORHEX was mixed with cyclohexane and deposited in a three-neck reactor with a condenser. Afterward, a solution of distilled water and APS purged by nitrogen was added. Thereafter, the reactor was hermetically closed and left stirring for 24 h protected from light. In the batch reaction, a single all compounds were added, and the synthesis was conducted for one period of 4 h. For the semicontinuous polymerization, the solution with surfactant ionic or nonionic, cyclohexane, water, and APS previously mixed was charged to the reactor. Followed the mixture of the aqueous phase of AHCl/water was added to the reactor using a B-D YALE 20 mL syringe adapted to a calibrated addition pump (Kd-Scientific) at a constant monomer addition rate (Ra) of 0.007 g/min for the microemulsion with AOT and a rate of 0.0152 g/min for the microemulsion with PEG-SORHEX. The process of semicontinuous addition was carried out for 1 h and the reaction continued for three additional hours. In the beginning, all reactions were placed in a temperature-controlled bath at 60°C with a stirring speed of 300 rpm and purged with N_2_ for 30 min to remove oxygen. The conversion was gravimetrically followed: in the microemulsion synthesized with AOT, a 5 ml aliquot was withdrawn in a time determined and placed in vials that were weighted previously. A mixture of water and ethanol (1:1 ratio) was added and the solution shook. The mixture and the vial were placed into an oven at 50°C for evaporate the solvent and precipitate the polymer. The polymer was filtered and washed several times with a water and ethanol mix to remove the AOT. The polymer and the vial were deposited in the oven to 50°C until obtained constant weight, and after conversions were determined by gravimetry. In the microemulsion synthesized with PEG-SORHEX, a 10 ml aliquot of the solution was withdrawn every determined time and placed in 50 mL centrifuge tubes of polypropylene and added acetone and centrifuged at 8500 RPM for 15 minutes. The centrifuge tube contained two phases, one phase in a solution and another polymer. The solution was extracted with a syringe, and acetone was added again. This process of separation was repeated three times. The polymer was withdrawn and was deposited into a glass vial of known weight and dried at 50°C until a constant weight was achieved.

### 2.3. Characterization

FTIR spectra of PANI samples were determined using Thermo Scientific iS5 Nicolet (Thermo Fisher Scientific, Madison, USA) with attenuated total reflection Fourier transform infrared spectroscopic (ATR-FTIR). The spectra were made at a 4 cm-1 resolution, and 32 scans. UV spectrograms were obtained by using a Genesys 10S UV.Vis brand UV-Vis spectrophotometer (Madison, WI, USA). Quartz cells were used. The calibrations were performed prior to the measurements with dimethyl sulfoxide. Latex particles of polyaniline (PANI) were examined in a JEOL 1010 Transmission Electron Microscope (Peabody, MA, USA) at a 100 kV voltage. The diameter and count of the particles were calculated by the program ImageJ 1.53. The descriptive statistic was realized by the program Origin 8.1. The diameters were obtained by measuring at least 50 particles in a micrograph. The diameter number-average size particle (D_n_), the diameter weight-average size particle (D_w_), the diameter z-average-particle size (D_z_), and polydispersity index (D_w_/ D_n_) were determined through the following equations:

(1) $$D_{n=}\frac{\sum_{n}^{i}n_{i}D_{i}}{\sum_{n}^{i}n_{i}}$$

(2) $$D_{w=}\frac{\sum_{n}^{i}n_{i}D_{i}^{4}}{\sum_{n}^{i}n_{i}D_{i}^{3}}$$

(3) $$D_{z=}\frac{\sum_{n}^{i}n_{i}D_{i}^{6}}{\sum_{n}^{i}n_{i}D_{i}^{5}}$$

where n_i_ is the number of particles, Di is the diameter of which particle and $\sum_{i}\;n_{i}$ is the total number of particles measured. The morphological analysis was conducted by a scanning electron microscope (SEM) TESCAN model MIRA3 (Warrendale, PA, USA). The conductivity tests were done in a Hioki LCR Meters model IM3570 (Shanghai, China). The value of conductivity was obtained in the frequency range of 1 kHz to 1000 kHz. Where an alternating perturbation from 1 V was applied to a 0.2 mm distance from the sample.

## 3. Results and discussion

### 3.1. Ternary diagram

**Figure 1.**
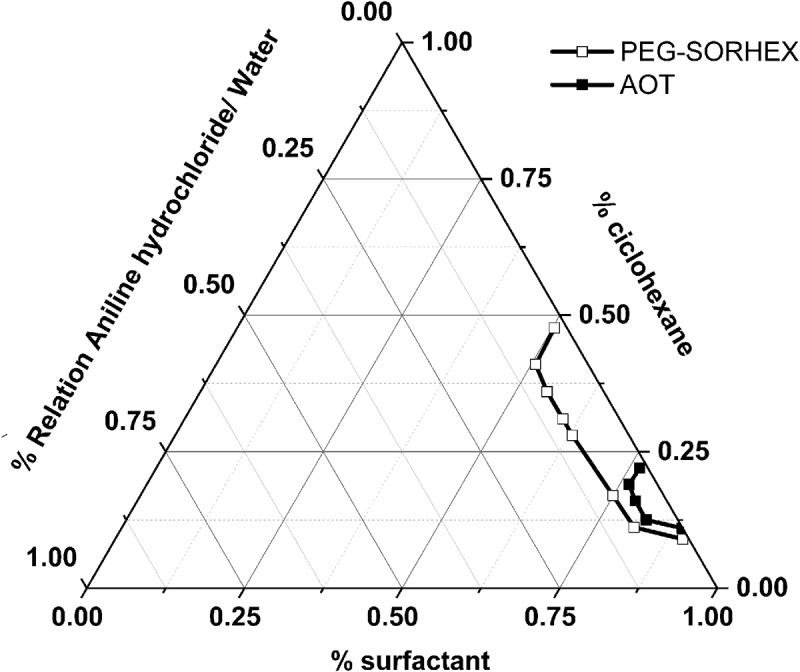
Phase diagram using ionic and nonionic surfactants.

Figure 1 shows the pseudoternary phase diagram of the system aniline hydrochloride-water/cyclohexane/ surfactant ionic (AOT) or nonionic (PEG-SORHEX) at 60°C temperature. The diagram shows the area from the microemulsion zone, which defined the path for the realization of the synthesis as a function of the components. Two reaction formulations were chosen that provided better stability and higher polymer conversion when synthesized PANI by batch and semi-continuous polymerization technique. The phase diagrams were made because there are few reports of articles where they present aniline hydrochloride/water ionic surfactant (AOT) [4,18] and in particular nonionic surfactant (PEG_SORHEX) in cyclohexane to form inverse microemulsion zones. As can be seen in the phase diagram, the capacity to solubilize aniline hydrochloride-water is rather limited and relatively few sensitive to the ionic surfactant in comparison to nonionic surfactant. The ability to solubilize aniline hydrochloride/water in ionic and nonionic surfactant systems will be limited by the radius of spontaneous curvature and the attraction between the droplets. Thus, for the aniline hydrochloride-water/AOT/cyclohexane system, the interactive attraction between the droplets decreases by making the layer more rigid due to the packing of the polar groups so that the degree of interpretation of the droplet is reduced and the reduction of the effect of curvature [19]. However, for the system where was used a nonionic surfactant such as PEG-SORHEX, the solubility of aniline hydrochloride-water is favored by the effects of the composition chemical of surfactant nonionic [20], which changed the radius of curvature.

### 3.2. Conversion

**Figure 2.**
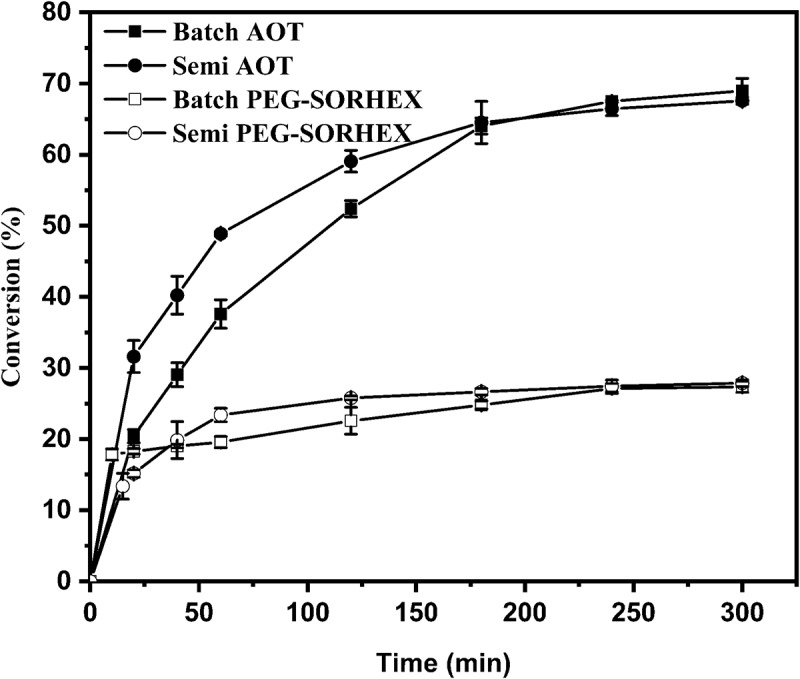
Conversion versus time for inverse microemulsion polymerization of PANI in batch and semicontinuous using surfactant ionic (AOT) and nonionic (PEG-SORHEX) at 60°C.

Figure 2 shows the conversion in batch and semi-continuous polymerization in inverse microemulsion for the different surfactants used. The graph of conversion against time shows the polymerization of the system with nonionic surfactant presents low conversion compared to the polymerization of the system with ionic surfactant. In the synthesis aniline hydrochloride molecules in the solution react with the oxidizing agent in the water, accelerating the diffusion process and improving the polymerization rate. Beginning of the reaction, the aniline hydrochloride is completely oxidized, when more aniline monomers are added, these are reduced and oxidized through the reaction medium until the oxidation potential is being low and the polymerization decreases [21]. Therefore, it can be deduced that the ionic surfactant was adequate to stabilize the aniline hydrochloride in water, due to the ionic strength and the polarizability of the polymerized particles. According to [22], the incorporation of a bulky anionic surfactant into polyaniline and polypyrrole prepared by chemical oxidative polymerization of monomers in an aqueous medium demonstrated an increase in the polymer yield. On the other hand, in reverse microemulsion where the surfactant was nonionic, the decrease in the conversion is due to various causes. One could be that the growth of the particles formed at low conversions is restricted by the very low concentration of monomer within the particles, and therefore the formation of new particles predominates over the swelling and growth of existing ones due to diffusion of monomers, a consequence of the amount and type of nonionic surfactant used [23]. Another cause is the effects of the nonionic surfactant, which caused the ionic strength to increase and the polymer formed with a positive charge to be affected by Van der Waals attractions or the very long aliphatic chains of the surfactant can be a steric barrier to oligomerization of the radical cations and later for the creation of polyaniline chains [22]. The results of [24, 25], ascribed the slower polymerization rate, to the presence of molecules of ethylene oxide into the structure of surfactants, ethylene oxide chain hinders the sulfate ions from oxidized into the anilinium cation. Other causes can be, the formation of the chemical environment, compartmentalization, the concentration of the reagents, the attraction or exclusion by electrostatic interaction by the Stern layer, and the oxidant concentration, promoted the formation of PANI with a higher content of soluble fraction of oligomers in the polar solvent [26–28]. Sapurina *et al*. [6] proposed that the formation of oligomers in the synthesis of PANI, due to the hydrolysis of the amino groups, generates the termination of the polymerization by the production of oligomers, thus blocking the continuation of the synthesis obtaining low conversions of polyaniline. However, determining the causes is left out of the study.

### 3.3. UV analysis

**Figure 3.**
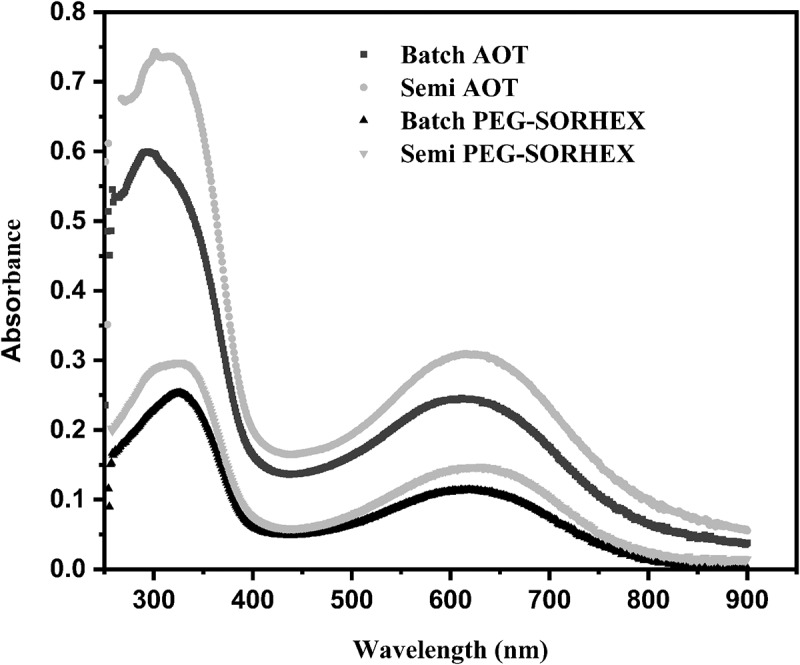
UV-Vis spectra of PANI in ionic and nonionic surfactant done by reverse microemulsion in batch and semicontinuous.

Figure 3 shows the spectra of UV visible of PANI synthesis by polymerization batch and semicontinuous realized with an ionic and nonionic surfactant. The graph shows two bands at 303–320 nm and 609–636 nm assigned to transition benzenoid and quinoid consistent with the molecular structure of the emeraldine base form. The first absorption band located at 303–320 nm is assigned to the π-π * transition in the benzenoid ring. The second absorption band located at 609–636 nm is assigned to the transition quinoid ring (charge transfer from HOMO of the benzenoid ring to LUMO of the quinoid ring) [29]. The ~600 nm signal is due to the formation of excitons in quinoid rings, these excitons are quasiparticles in which there are Coulombic interactions between an electron and a vacancy of opposite charge and are only found in semiconductor or insulating materials [30]. These bands provide information on the general oxidation state of PANI. For determining the degree oxidation, the spectra of UV-Vis were normalized by baseline and were calculated the intensity by the origin lab 8 program, the ratio is calculated as follows:

(4) $$R\left(Intensity\;ratio\right)=\frac{I_{630}}{I_{330}}$$

where I is the intensity absorption band. The values of R = 0.9307 and 0.8251 for PANI synthesized by batch/AOT and semicontinuous/AOT were determined, respectively. For PANI synthesized by batch/PEG-SORHEX and Semicontinuous/PEG-SORHEX, the values were 0.8050 and 0.7439. For each case, values less than 1 indicate that there are more benzene units within the polymer, and values of 1 define structure type emeraldine and the polymer presents more conductivity [31]. In this case, the value oxidation circa to one was for PANI synthesized by AOT by batch and semicontinuous. This suggests that the surfactant ionic (AOT) used in the synthesis was one important parameter that affects the oxidation state of PANI. Table 1 depicts the results of oxidation degree, conversion percentage, and conductivity. The conductivity and the yield of polyaniline synthesized were found to depend on the kind of surfactant (Table 1). The yield of the product increased when the synthesis was realized with surfactant ionic (AOT) in comparison to surfactant nonionic (PEG-SORHEX). The conductivity of polyaniline synthesized by the semicontinuous process at 60°C was higher up to three orders of magnitude compared to that prepared at the same temperature by the batch process for both kinds of surfactant. According to 32, the increase of the conductivity is due to the homogeneous protonation of the imine nitrogen and good conformational ordering. This depends on the synthesis, the dopant, the surfactant, and the concentration of reactants.

**Table 1.** Effects of the reaction of polyaniline in the yield, oxidation degree, and conductivity

Samples	yield (%)	I_Q/B_^a^	σ (S/cm)
Batch/ AOT	67.5	0.9307	1.3x10^−10^
Semi/ AOT	66.4	0.8521	7.7X10^−7^
Batch/ PEG-SORHEX	27.1	0.8050	1.1x10^−7^
Semi/ PEG-SORHEX	27.4	0.7439	2.7x10^−4^

^a^I_Q/B_ is a relation quinoid (Q)/ benzenoid (B).

### 3.4. FTIR analysis

**Figure 4.**
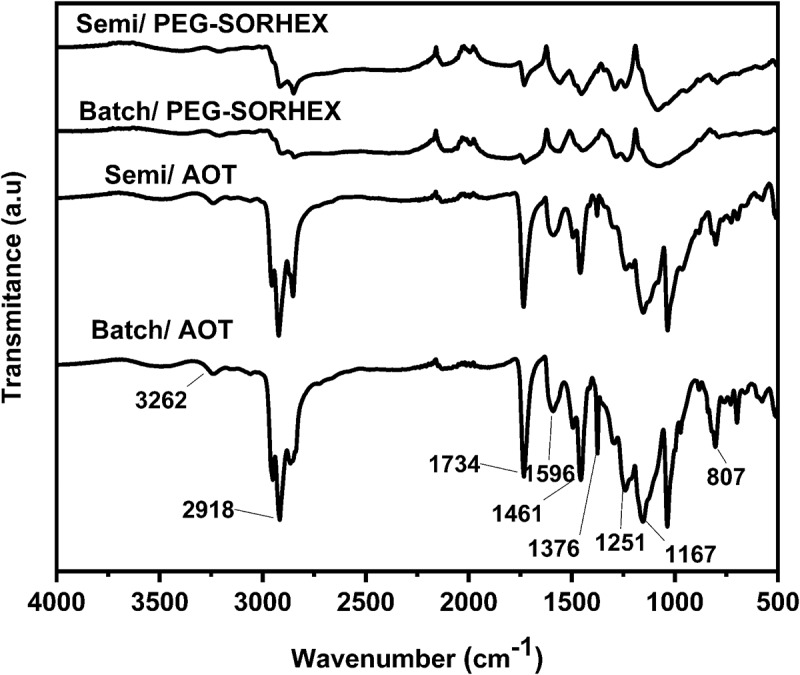
FTIR spectra of PANI synthesized in batch and semicontinuous using surfactant ionic and nonionic.

The FTIR spectrum of PANI is shown in Figure 4. The 3262 cm^−1^ peak is attributed to N-H stretching mode, and the 688 cm-1 peak is attributed to N-H movement. IR spectra of PANI show the main characteristic peaks of the samples at 1596 cm^−1^, 1461 cm^−1^, 1376 cm^−1^, 1251 cm^−1^, and 807 cm^−1^ in agreement with the literature [33]. The spectrum shows two bands in the region of 1596 cm^−1^, and 1461 cm^−1^ corresponding to the stretching vibrations of the quinoid (Q) and benzenoid (B) rings of polyaniline. A band near 1376 cm^−1^ is C‚N+ stretching adjacent to the quinoid structure, while a band at 1306 cm^−1^ is associated with C–N stretching vibration in alternating quinoid-benzenoid-quinoid ring units as shown in Figure 4. The peaks at 1376 cm^−1^ and 1251 cm^−1^ correspond to the N-H bending and the symmetric component of the C–C or C–N stretching modes. The delocalization of π electrons induced by polymer protonation is related to the bands at 1232–1250 cm^−1^ that are vibration signals in the C-N+ · polaron structure. These signals are more defined in the spectra corresponding to the polymers that showed higher conductivity, such as those synthesized with PEG-SORHEX [34]. The peaks at 1151–1162 cm^−1^ are vibration signals of charged polymers, either Q = NH+·-B or B-NH+·-B indicating the presence of charges in the polymer chain. The bands corresponding to 792 and 815 cm^−1^ are due to out-of-plane vibrations in 1,4-substituted aromatic rings. On the other hand, the bands from 2841 to 2968 cm^−1^ are attributed to signals caused by stretching of the C-H bond [35]. The band around 1167 cm^−1^ is attributed to the benzenoid–(NH+) ‚ and quinoid structure that forms during the protonation process. The 1596 cm^−1^ peak confirms the presence of a protonated imine, and the 1251 cm^−1^ peak is characteristic of the conduction of a protonated form of PANI [27,29]. In the case of the spectra of samples synthesized with active surface PEG-SORHEX, some different signals can be found from the spectra of the PANI synthesized with active surface AOT, in the bands of 860 cm^−1^ and 943 cm^−1^ that indicate signals of 1,2,4 trisubstituted rings [36] this coincides with the structure of phenazine. The formation of trimers with a phenazine structure functions as initiation centers for the growth of polyaniline chains that have reactive sites in para positions [6].

### 3.5. TEM analysis

**Figure 5.**
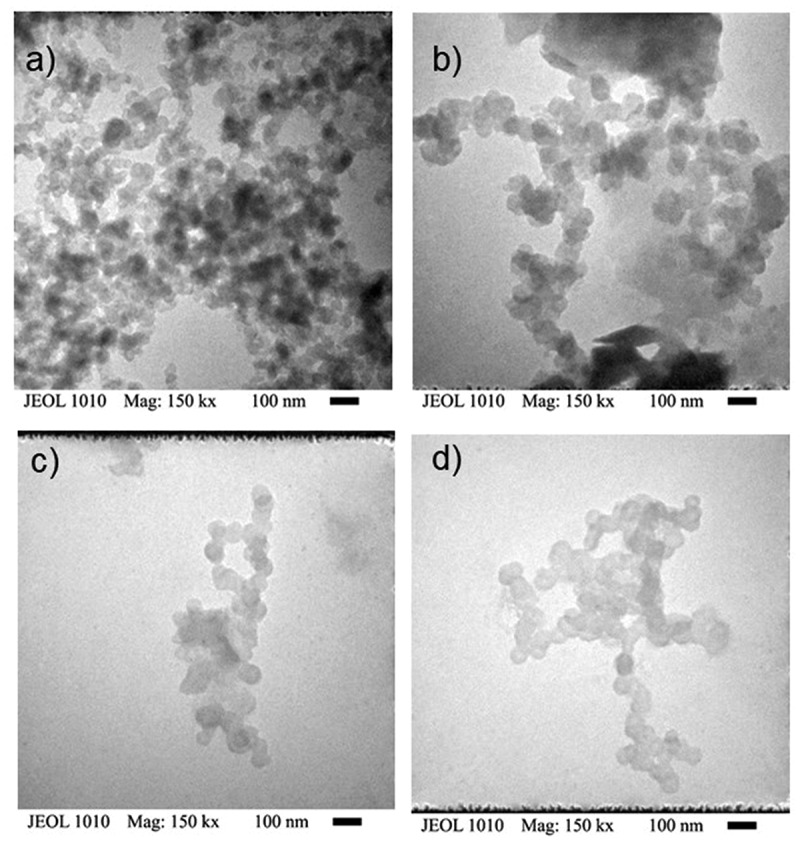
TEM images of PANI synthesized by a) Batch AOT b) Semi AOT, c) Batch PEG-SORHEX and d) Semi PEG-SORHEX.

Figure 5 shows micrographs of PANI synthesized in batch and semicontinuous using surfactant ionic (AOT) and nonionic (PEG-SORHEX). In all cases, the micrographs show quasi-spherical particles; this is due to the deformation by heating due to the electron beam when examining in TEM. The results from diameters of the particle of the latex of PANI are reported in Table 2. Table 2 shows that the latexes contain a nearly mono-dispersed population of particles (1.05 < Dw/Dn < 1.29). The polydispersity diminishes when used the process of polymerization in batch in comparison to the polymerization semicontinuous. This is an indication that the control of the addition of monomers affects polydispersity. The values of the diameters calculated in the synthesis by ionic surfactant are smaller than synthesized by a nonionic surfactant in the different processes done, because nonionic surfactant decreases the effective packing parameter promoted in part by the hydrophilicity of the surfactant causing the droplet size to increase for the ionic system [16]. Regarding the process of polymerization, it is observed that the diameter particle synthesized in semicontinuous is bigger than the synthesized in batch, due to the control of monomer addition in the system, and this influences the distribution and particle size of the latex [37].

**Table 2.** Diameter particle of PANI synthesized by batch and semicontinuous

Samples	D_n_ (nm)	D_z_ (nm)	D_w_/D_n_
Batch AOT	36.25	44.83	1.15
Semi AOT	48.88	70.16	1.29
Batch/PEGSORHEX	55.29	59.18	1.04
Semi/PEGSORHEX	73.96	96.96	1.23

### 3.6. SEM image analysis

**Figure 6.**
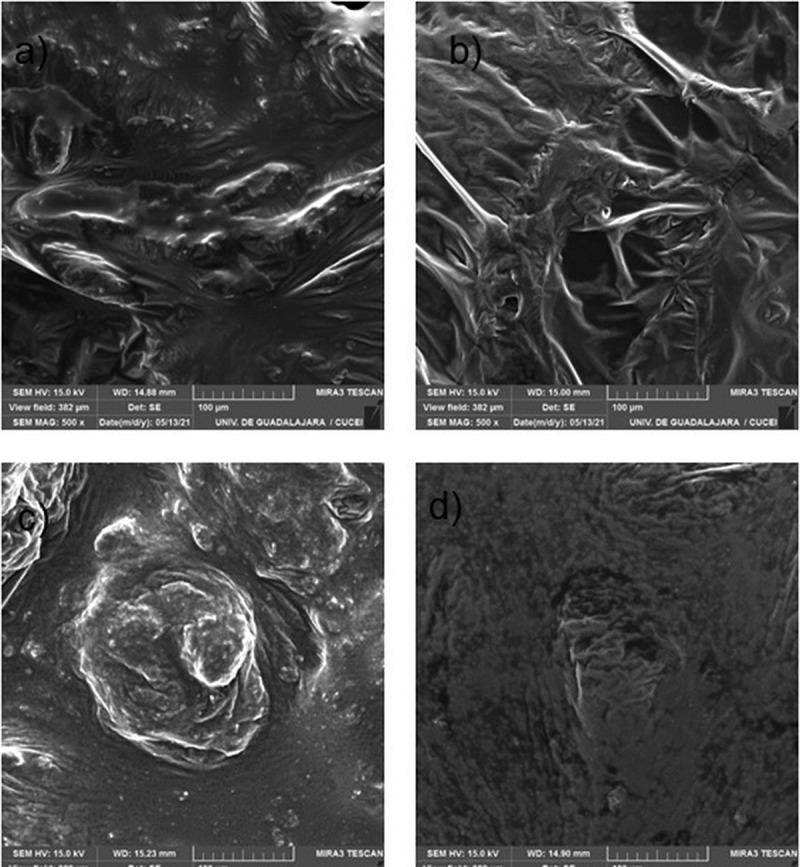
Scanning electron microscope micrograph of PANI synthesis: a) Batch/ AOT b) Semicontinuous/AOT, c) Batch/ PEG-SORHEX and d) Semicontinuous/ PEG-SORHEX.

Figure 6 shows SEM micrographs of PANI synthesized with ionic surfactant (AOT) and nonionic surfactant (PEG-SORHEX) by process batch and semicontinuous. In Figure 6(a,b), the morphology of PANI synthesized with ionic surfactant by the discontinuous process and the semicontinuous process is observed, and the morphology changes from agglomerate to agglomerate with a bar. This is due to the addition of water/monomer into the micellar system, and how it is fed the water/monomer to the system by batch and semicontinuous processes, this increases the intermicellar exchange of matter between the AOT-inverted micelles. This exchange induces one-dimensional growth and ultimately aggregates formation through intermicellar collisions [14]. According to 24,38 and, the morphology of PANI particles can change depending on the synthesis method, the types of surfactants, the nature of the oxidizing agent, and reaction conditions. When the surfactant is removed, the PANI nanoparticles can be regularly arranged to form a granular agglomerate morphology due to the small size of the nanoparticles [14]. Figures 6(c,d) show the micrographs of PANI made with nonionic surfactant (PEG-SORHEX) by discontinuous process and semicontinuous process, and the morphology changes from agglomerates to agglomerates with a dendritic structure. When the nonionic surfactant was removed, the PANI nanoparticles can organize regularly to form a granular agglomerate morphology, caused by the conductive nanoparticles forming structures or agglomerates of nanoparticles that have very strong affinities with each other, which are grouped during the preparation of the sample, obtaining different morphologies., Caused by the nonionic surfactant within the inverse microemulsion that favors the packing parameter, forming spherical micelles due to the presence of oxyethylene groups [39]. Different investigations in heterogeneous polymerization attribute the formation of the different morphologies to the self-assembly behavior of nonionic surfactants that form micelles in the organic phase induced by the environment and the addition of monomers in the PANI polymerization [40–42].

## 4. Conclusions

Optimum synthesis conditions for the polymerization in microemulsion of aniline by ammonium persulphate oxidation in batch process and semicontinuous process using surfactant ionic and nonionic was established. The yield was higher for the ionic surfactant compared to the nonionic. Spectroscopic studies of UV-Vis showed a ratio of the relative intensities of the quinoid to benzenoid unit closed to one. The conductivity of polyaniline synthesized by polymerization semicontinuous process is higher up to three orders of magnitude compared to the polymerization batch process for both surfactants. Different PANI nanostructures were obtained by SEM. The formation of the different morphologies is due to the self-assembly behavior of surfactant ionic and nonionic that form micelles in the phase organic induced by the environment and the addition of aniline hydrochloride/water in the polymerization. The results of TEM show the particle size synthesized in polymerization semicontinuous process is bigger than the ones synthesized in a process polymerization batch process.

## References

[1] Belaabed B, Lamouri S, Naar N, et al. Polyaniline-doped benzene sulfonic acid/epoxy resin composites: structural, morphological, thermal and dielectric behaviors. Polym J. 2010;42(7):546–554.

[2] Šeděnková I, Konyushenkoa EN, Stejskal J, et al. Solid-state oxidation of aniline hydrochloride with various oxidants. Synth Met. 2011;161(13–14):1353–1360.

[3] Chowdhury P, Saha B. Potassium dichromate initiated polymerization of aniline. Indian J Chem Technol. 2005;12:671–675.

[4] Xia H, Wang Q. Synthesis and characterization of conductive polyaniline nanoparticles through ultrasonic assisted inverse microemulsion polymerization. J Nanopart Res. 2001;3(5/6):401–411.

[5] Gusain M, Nagarajan R, Singh SM. Highly ordered polyaniline: synthesis, characterization, and electrochemical properties. Polym Bull. 2020;77(6):3277–3286.

[6] Sapurina I, Stejskal I. The mechanism of the oxidative polymerization of aniline and the formation of supramolecular polyaniline structures. Polym Int. 2008;57(12):1295–1325.

[7] Tang SJ, Wang AT, Lin SY, et al. Polymerization of aniline under various concentrations of APS and HCl. Polym J. 2011;43(8):667–675.

[8] Shreepathi S, Holze R. Spectro electrochemical investigations of soluble polyaniline synthesized via new inverse emulsion pathway. Chem Mater. 2005;17(16):4078–4085.

[9] Zhang X, J Zhu J, Haldolaarachchige N, et al. Synthetic process engineered polyaniline nanostructures with tunable morphology and physical properties. Polymer. 2012;53:109–2120.

[10] Alvarado AG, Nolla J, Rabelero M, et al. Poly(hexyl methacrylate) nanoparticles templating in nanoemulsions-made by phase inversion temperature. J Macromol Sci, Part A Pure Appl Chem. 2013;50(4):385–39. 10.1080/10601325.2013.768119.

[11] Chasana NU, Ulfa SM, Masruri M. Synthesis and characterization of polyaniline nanoparticle by inverse micelle microemulsion method. J Pure App Chem. 2017;3(3):189–195.

[12] Ovando-Medina VM, Piña-García PS, Corona-Rivera MA, et al. Semicontinuous heterophase polymerization of methyl methacrylate in the presence of reactive surfactant HITENOL BC10. Polym Bull. 2012;68(9):2313–2322.

[13] Suna P, Misra PK. Effect of ionic and nonionic surfactants on the phase behaviour and physicochemical characteristics of pseudoternary systems involving polyoxyethylene(20)sorbitan monooleate. Surf Interfaces. 2018;10:19–26.

[14] Zhou Q, Wang J, Ma Y, et al. The relationship of conductivity to the morphology and crystallinity of polyaniline controlled by water content via reverse microemulsion. Colloid Polym Sci. 2007;285(4):405–411.

[15] Ichinohe D, Arai T, Kise H. Synthesis of soluble polyaniline in reverse micellar systems. Synth Met. 1997;84(1–3):75–76.

[16] Liu J, Zhang X, Zhang H. Water/AOT/IPM/alcohol reverse microemulsions: influence of salts and nonionic surfactants on structure and percolation behavior. J Chem Thermodyn. 2014;72:1–8.

[17] Hensel JK, Carpenter AP, Ciszewski RK, et al. 2017. Molecular characterization of water and surfactant AOT at nanoemulsion surfaces. Proceedings of the National Academy of Sciences, USA, 114, 13351–13356 10.1073/pnas.1700099114 PMC5754752 28760977

[18] Selvan ST, Mani A, Athinarayanasamy K, et al. Synthesis of crystalline polyaniline. Mater Res Bull. 1995;30(6):699–705.

[19] Hou MJ, Shah DO. Effects of the molecular structure of the interface and continuous phase on solubilization of water in water/oil microemulsions. Langmuir. 1987;3(6):1086–1095.

[20] Florence AT, Rogers JA. Emulsion stabilization by non-ionic surfactants: experiment and theory. J Pharm Pharmac. 1971;23(3):153–169. 10.1111/j.2042-7158.1971.tb08637.x 4397235

[21] Marie E, Rothe R, Antonietti M, et al. Synthesis of polyaniline particles via inverse and direct miniemulsion. Macromolecules. 2003;36(11):3967–3973.

[22] Stejskala J, Omastova M, Fedorovac S, et al. Polyaniline and polypyrrole prepared in the presence of surfactants: a comparative conductivity study. Polymer. 2003;44(5):1353–1358.

[23] Aguilar J, Rabelero M, Nuño-Donlucas SM, et al. Narrow size-distribution poly(methyl methacrylate) nanoparticles made by semicontinuous heterophase polymerization. J Appl Polym Sci. 2011;119(3):1827–1834.

[24] Kim BJ, Oh SG, Han MG, et al. Synthesis and characterization of polyaniline nanoparticles in SDS micellar solutions. Synth Met. 2001;122(2):297–304.

[25] Kohut-Svelko N, Reynaud S, Francois J. Synthesis and characterization of polyaniline prepared in the presence of nonionic surfactants in an aqueous dispersion. Synth Met. 2005;150(2):107–114.

[26] Cao Y, Andreatta A, Heeger AJ, et al. Influence of chemical polymerization conditions on the properties of polyaniline. Polymer (Guildf). 1989;30(12):2305–2311.

[27] Mazzeu MAC, Faria LK, Baldan MR, et al. Influence of reaction time on the structure of polyaniline synthesized on a pre-pilot scale. Brazilian J Chem Eng. 2018;35(1):123–130.

[28] Oh SG, Kizling J, Holmberg K. Microemulsions as reaction media for synthesis of sodium decyl sulfonate 2. role of ionic surfactants. Colloids Surf A Physicochem Eng Asp. 1995;104(2–3):217–222.

[29] Ibrahim KA. Synthesis and characterization of polyaniline and poly(aniline-co-o-nitroaniline) using vibrational spectroscopy. Arab J Chem. 2017;10:S2668–S2674.

[30] Gul S, Shah AUHA, Bilal S. Synthesis and characterization of processable polyaniline salts. J Phys Conf Ser. 2013;439:1.

[31] Abdiryim T, Xiao-Gang Z, Jamal R. Comparative studies of solid-state synthesized polyaniline doped with inorganic acids. Mater Chem Phys. 2005;90:367–372.

[32] Rao PS, Sathyanarayana DN, Palaniappan S. Polymerization of aniline in an organic peroxide system by the inverted emulsion process. Macromolecules. 2002;35(13):4988–4996.

[33] Plesu N, Ilia G, Bandur G, et al. Chemical polymerization of aniline in phenylphosphinic acid. J Serbian Chem Soc. 2005;70(10):1169–1182.

[34] Ping Z. In situ FTIR–attenuated total reflection spectroscopic investigations on the base–acid transitions of polyaniline. Base–acid transition in the emeraldine form of polyaniline. J Chem Soc, Faraday Trans. 1996;92(17):3063–3067.

[35] Saravanan S, Mathai CJ, Anantharaman MR, et al. Investigations on the electrical and structural properties of polyaniline doped with camphor sulphonic acid. J Phys Chem Solids. 2006;67(7):1496–1501.

[36] Trchová M, Stejskal J. Polyaniline: the infrared spectroscopy of conducting polymer nanotubes (IUPAC Technical report). Pure Appl Chem. 2011;83(10):1803–1817.

[37] Lovel PA, El-Aasser MS. Emulsion polymerization and emulsion polymers. 1997 1 t editio. Chichester(EN): John Wiley & Sons, Inc. 9780. 9780471967460471967460

[38] Bhadra S, Singha NK, Khastgir D. Electrochemical synthesis of polyaniline and its comparison with chemically synthesized polyaniline. J Appl Polym Sci. 2007;104(3):1900–1904.

[39] Kumar S, Singh V, Aggarwal S, et al. Synthesis of polyaniline nanostructures via reverse microemulsion technique. Soft Mater. 2009;7(3):150–163.

[40] Calheiros LF, Soares BG, Barra GMO. DBSA-CTAB mixture as the surfactant system for the one step inverse emulsion polymerization of aniline : characterization and blend with epoxy resin. Synth Met. 2017;226:139–147.

[41] Freitas TV, Sousa EA, Fuzari GC, et al. Different morphologies of polyaniline nanostructures synthesized by interfacial polymerization. Mater Lett. 2018;224:42–45.

[42] Yang L, Wu W, Ohki Y, et al. Enhanced conductivity of polyaniline in the presence of nonionic amphiphilic polymers and their diverse morphologies. J Appl Polym Sci. 2017;134(47):1–10.

